# SEPDB: a database of secreted proteins

**DOI:** 10.1093/database/baae007

**Published:** 2024-02-12

**Authors:** Ruiqing Wang, Chao Ren, Tian Gao, Hao Li, Xiaochen Bo, Dahai Zhu, Dan Zhang, Hebing Chen, Yong Zhang

**Affiliations:** The State Key Laboratory of Complex, Severe, and Rare Diseases, Institute of Basic Medical Sciences, Chinese Academy of Medical Sciences and School of Basic Medicine, Peking Union Medical College, #5 Dong Dan San Tiao, Beijing 100005, China; Experimental Center, Shandong University of Traditional Chinese Medicine, Key Laboratory of Traditional Chinese Medicine Classical Theory, Ministry of Education, Shandong University of Traditional Chinese Medicine, #4655 Daxue Road, Changqing District, Jinan, Shandong Province 250355, China; Institute of Health Service and Transfusion Medicine, #27 Taiping Road, Haidian District, Beijing 100850, China; The State Key Laboratory of Complex, Severe, and Rare Diseases, Institute of Basic Medical Sciences, Chinese Academy of Medical Sciences and School of Basic Medicine, Peking Union Medical College, #5 Dong Dan San Tiao, Beijing 100005, China; Experimental Center, Shandong University of Traditional Chinese Medicine, Key Laboratory of Traditional Chinese Medicine Classical Theory, Ministry of Education, Shandong University of Traditional Chinese Medicine, #4655 Daxue Road, Changqing District, Jinan, Shandong Province 250355, China; Institute of Health Service and Transfusion Medicine, #27 Taiping Road, Haidian District, Beijing 100850, China; Institute of Health Service and Transfusion Medicine, #27 Taiping Road, Haidian District, Beijing 100850, China; The State Key Laboratory of Complex, Severe, and Rare Diseases, Institute of Basic Medical Sciences, Chinese Academy of Medical Sciences and School of Basic Medicine, Peking Union Medical College, #5 Dong Dan San Tiao, Beijing 100005, China; Bioland Laboratory (Guangzhou Regenerative Medicine and Health Guangdong Laboratory), #96 South Xingdao Ring Road, Haizhu District, Guangzhou 510005, China; Experimental Center, Shandong University of Traditional Chinese Medicine, Key Laboratory of Traditional Chinese Medicine Classical Theory, Ministry of Education, Shandong University of Traditional Chinese Medicine, #4655 Daxue Road, Changqing District, Jinan, Shandong Province 250355, China; Institute of Health Service and Transfusion Medicine, #27 Taiping Road, Haidian District, Beijing 100850, China; The State Key Laboratory of Complex, Severe, and Rare Diseases, Institute of Basic Medical Sciences, Chinese Academy of Medical Sciences and School of Basic Medicine, Peking Union Medical College, #5 Dong Dan San Tiao, Beijing 100005, China; Bioland Laboratory (Guangzhou Regenerative Medicine and Health Guangdong Laboratory), #96 South Xingdao Ring Road, Haizhu District, Guangzhou 510005, China

## Abstract

Detecting changes in the dynamics of secreted proteins in serum has been a challenge for proteomics. Enter secreted protein database (SEPDB), an integrated secretory proteomics database offering human, mouse and rat secretory proteomics datasets collected from serum, exosomes and cell culture media. SEPDB compiles secreted protein information from secreted protein database, UniProt and Human Protein Atlas databases to annotate secreted proteomics data based on protein subcellular localization and disease markers. SEPDB integrates the latest predictive modeling techniques to measure deviations in the distribution of signal peptide structures of secreted proteins, extends signal peptide sequence prediction by excluding transmembrane structural domain proteins and updates the validation analysis pipeline for secreted proteins. To establish tissue-specific profiles, we have also created secreted proteomics datasets associated with different human tissues. In addition, we provide information on heterogeneous receptor network organizational relationships, reflective of the complex functional information inherent in the molecular structures of secreted proteins that serve as ligands. Users can take advantage of the Refreshed Search, Analyze, Browse and Download functions of SEPDB, which is available online at https://sysomics.com/SEPDB/.

**Database URL:**  https://sysomics.com/SEPDB/

**Key Points**

- In its inception, SEPDB is a secreted protein database created by collating experimentally validated secreted protein datasets and predicted secreted proteins *in silico*.
- In application, SEPDB is a large-scale integrated platform for annotation and analysis of secreted proteins that reveals correlations between expression levels and functions of secreted proteins under different conditions, such as exercise, aging and disease.

## Introduction

Secretory proteins, produced by secretory cells, including immune B cells, exert autocrine, paracrine or endocrine effects (1). Secreted proteins, which can take the form of antibodies, enzymes, cellular chemokines and various metabolite components, are specifically transported from the intracellular to the extracellular space in various tissues to activate specific functional pathways; as such, they can be useful in the diagnosis and treatment of the disease (2). The mechanisms governing the regulation of secreted proteins and their interaction with organs provide insight into the relationship between protein function and disease. The release of secreted proteins originating from secretory cells of individual tissues into the serum initiates the communication-signal function of these proteins. This secretory function affects cellular metabolism through transcriptomic and proteomic changes; as new secretory factors are discovered, they contribute to target discovery or drug development and help explain genomic variation (3). Secretory proteomics is an ‘omics’ field, which explores the transcriptional regulation of the genome, the principles of kinase activation and inhibition and the mechanisms of metabolite action (4). Secretory proteomics identifies molecular communication networks for tissues (e.g. adipose tissue and bone) and organs (e.g. liver, pancreas and brain). Protein analysis methods, including mass spectrometry, liquid chromatography-mass spectrometry, protein microarray technology and other rapidly emerging techniques, combine to enrich the ability to mine secretory proteomics data and provide datasets of secretory proteins from various model organisms (5).

Serum proteomics is an invaluable tool for collecting information on changes in exosomes and secreted proteins in different physiological or disease states, given the utility of these changes as biomarkers and therapeutic targets for different types of diseases (6). Secretory proteomics can identify and validate drug targets, ultimately providing new drugs and therapeutic options for the treatment of the disease (7). Secretory proteomics can also serve as a novel indicator for treatment and prognosis, as well as a valuable tool for uncovering the mechanism of action of new drugs. Significant changes in the composition of protein biomarkers can be used to assess the effect of a particular drug intervention (2). Analyzing secretory proteomics and transcriptomic expression data across various tissues helps unearth the source and target organs of secretory proteins. Secretory proteomics research aims to identify and characterize potential biomarkers through the interpretation of protein structure and function. By analyzing metabolic components in serum after the targeted knockdown of specific genes, advanced proteomics techniques provide protein functional information, seamlessly integrating tissue-specific information derived from transcriptomic data (8). Molecular studies have shown, for example, that exercise modulates the metabolic disorders induced by a high-fat diet, demonstrating that skeletal muscle contraction stimulates the body’s energy production, increases cellular activity and releases secretory proteins that promote glucolipid metabolism and improve resistance to obesity (9). Metabolic disorders associated with aging are correlated with a decrease in the quality of protein molecules released into the serum and an increase in the expression of inflammatory and tumor factors, concurrent with an age-related decrease in body mass (10).

The detection of secretory proteins in serum is limited by their low abundance, insufficient annotation and the lack of research methods for collecting information on these proteins. These limitations are highlighted by a hierarchy of specific issues. (i) Normalization of secretory proteomics data is complex and does not allow accurate annotation of the mechanisms of action of secretory proteins. (ii) Secretory proteins come in a variety of forms, including antibodies, digestive enzymes, exosomes and metabolic molecules. Navigating this diversity is made more difficult by the absence of a structured initiative to distinguish secretory proteins based on the collation of validated literature data on the subject and the paucity of data and regulatory studies on secretory proteins from different model organisms. (iii) Secretory proteins that act as ligands bind specifically to cellular receptors in target tissues, forming a cellular communication network that coordinates the intracellular environment and maintains metabolic homeostasis. Extracting network-level information from the specificity of secretory protein-receptor expression, which varies depending on tissue-specific dynamics, has proven to be exceptionally complex. (iv) Signal peptide prediction and transmembrane structural domain models are continuously being updated, but efforts to systematically collate information on secretory protein signal peptides and structural extensions of transmembrane domains have not kept pace with their development. Moreover, existing models often predict secretory proteins according to varying standards and are subject to interference from transmembrane structural signal peptides, resulting in unacceptably high false-positive rates. (v) The redundancy of information on proteins from different model organisms makes it challenging to cross-reference specific secreted proteins. For all of these reasons, we developed secreted protein database (SEPDB) to aggregate tissue-specific secretory proteomic information, interpret organ or tissue interactions and cross-validate proteins from serum, exosomes and tissue culture supernatants with predicted data. SEPDB aims to capture and validate the origin of secretory proteins through experimental evidence and add to the predictive secretome data.

## Materials and methods

### Data collection

**Figure 1.**
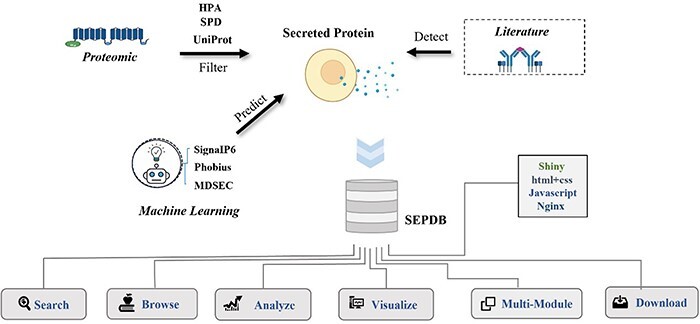
Flowchart of SEPDB construction. SEPDB collects and processes relevant protein data from several databases and manually adds literature data from serum, exosomes and tissue culture to predict large-scale amino acid sequences. These data are used to implement complex web interfaces.

The secretory proteomics dataset is derived from three main data sources, as shown in Figure 1: (i) secreted protein data from literature on serum, exosomes and tissue culture media, as exemplified by the report by Katrin *et al*. on the *ex vivo* enrichment of secreted proteins by click chemistry and their pulsed stable isotope labeling in combination with amino acids in cell culture (11). (ii) Datasets from secreted protein database (SPD), Human Protein Atlas (HPA) and UniProt databases (12). (iii) SignaIP6.0 predicts the signal peptide structure of proteins as secreted in the database, and it ensures that no obvious membrane proteins are incorrectly categorized as structural secretory proteins (13). Phobius and a framework for prediction and evaluation of secreted proteins (SPOCTOPUS) prediction models are included to detect reference data, reduce false-positive validation of transmembrane structural domains as signal peptides and provide a basis for the identification of secreted proteins (14, 15). Data from rats and mice provide further relevant information for protein annotation (16, 17).

SEPDB integrates datasets from the literature (e.g. serum, exosomes and tissue cultures), subcellular localization datasets from a priori databases and datasets calculated by models. SEPDB also organizes modules for the aging-related secreted proteins dataset and the exercise-related secreted proteins dataset.

### Data annotation

**Figure 2.**
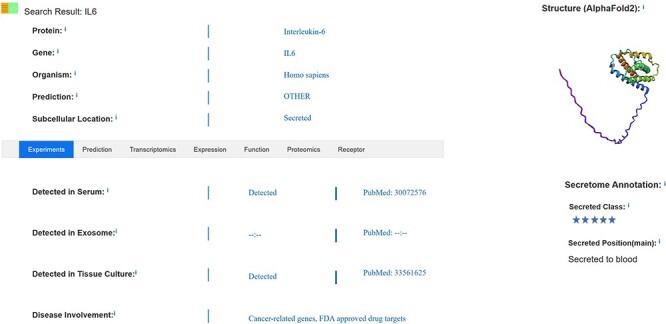
SEPDB result page.

Basic information about a protein, such as its official protein name, corresponding gene name, protein secretion class, secretion site and secretion-specific organization, among others, is annotated. Information on detection of the protein in exosomes, serum and tissue culture supernatants is indicated, as shown in Figure 2; information on tissue-specific expression in different physiological states is annotated; information on the loci of the secreted protein in HPA and UniProt databases is screened; and information on whether the protein is a marker for certain diseases is interpreted. Evidence for detection in plasma, exosomes and tissue culture media is manually annotated through literature searches. Some of the secreted proteins have been annotated with multiple disease markers based on subcellular localization, which capture data on extra-membrane proteins (2, 7). SEPDB aggregates information from multiple protein databases and annotates both signal peptide data and transmembrane structural domain information to improve the screening criteria for secreted proteins. Specific secretory tissues are annotated according to the transcriptomic and proteomic heterogeneity of secreted proteins in different tissues, detailed expression values in each tissue are provided, and transcriptomic and protein data for that tissue with high expression are selected for presentation (8, 18).

After annotation of basic information on secreted proteins, data based on detection in serum, exosomes or tissue culture media are manually collected and compared with data defined as secreted by subcellular localization in the UniProt database. These pre-selected secreted proteins are further processed using the Signal Peptide Prediction Tool, and datasets are further validated through the Genotype-Tissue Expression transcriptomics database and proteomics annotation. Some disease markers are annotated from the HPA, and this dataset is expanded through addition of biomarkers annotated in the literature. Non-transmembrane domain proteins have signal peptide structure, which is the basis for identification as secreted proteins (19).

In the SEPDB dataset, we score secreted protein datasets. Evidence for secreted proteins is categorized into five levels, with higher scores being more reliable. The five-score datasets are secreted proteins from the literature (e.g. serum, exosomes and tissue culture fluids) that have been validated in a priori databases (e.g. UniProt, HPA, etc.) and have also passed the model predictions of signal peptides. Four-score datasets are those available in the literature and validated in other databases. The three-score dataset is available in the literature and calculated in the model. The two-score dataset is data documented with evidence in the literature. One-score datasets are results of model calculations or evidence of data in other databases.

### Secretory protein-receptor network

SEPDB not only focuses on exploring the origin of secreted proteins and collating changes in the expression of genes encoding secreted proteins, it also integrates information on the cognate receptors of secreted protein ligands and constructs receptor expression networks based on protein interactions, incorporating highly relevant receptor information obtained using CellChat and CellPhoneDB tools (20, 21). Utilizing cellular communication data, users have the option of viewing information on the tissue specificity of the target receptor for the secreted protein ligand; moreover, information on the target and the secretion pathway of the secreted protein can be acquired through receptor transcriptomics and proteomics correlation data (22, 23).

### Prediction of secreted proteins

Predictions of secreted proteins are based on predicted N-terminal signal peptides and transmembrane regions, with additional predictive insight contributed by subcellular location information provided by UniProt. Signal peptides are present in most secreted proteins but are also found in certain types of transmembrane proteins, which complicate the prediction and identification of secreted proteins. In tests against existing protein amino acid databases, four signal peptide prediction methods—SignaIP6.0, Phobius, SPOCTOPUS and a secretory protein prediction model (MDSEC)—were all shown to be reliable (14, 15). Signal peptides, characterized by their short amino acid sequences and sequence regularity, can be reliably predicted. SignaIP6.0 is notable for its ability to detect types of signal peptides but also shows slightly greater accuracy in predicting signal peptides and capturing a broader range of amino acid sequences containing signal peptides compared with other prediction tools. However, it should be noted that SignaIP6.0 also shows an extremely high false-positive rate for transmembrane structural domain signal peptides (13).

We have taken advantage of the model’s ability to annotate tens of thousands of unannotated protein sequences to update the secreted protein dataset by incorporating SignaIP6.0. This updated model can predict structural sequences of signal peptides that could not be detected by previous versions. To further optimize the SEPDB core dataset, we retrained the model on the core dataset. To address the issue of the robustness of SignaIP6.0 and the confounding effects of transmembrane domain topology, we predicted the SEPDB core dataset using the Phobius hidden Markov model. This model combines transmembrane topology and signal peptide prediction, improving the already high accuracy of the transmembrane hidden Markov model in predicting transmembrane structural domains and reducing the false-positive rate of transmembrane proteins. We further authenticated the core protein dataset with high accuracy using a Phobius overlap analysis of signal peptide and transmembrane segment predictions (14). SEPDB compiles prediction results from SignaIP6.0, Phobius, MDSEC and other models; redefines the core set of secretory proteins; hierarchically classifies them; compares their prediction with proteomic identification data; captures bona fide secretory proteins from among predicted secretory proteins; and extends their boundaries through model predictions.

### Secreted proteins in the senescence module

Secreted proteins play a crucial role in physiological, developmental and disease processes, including aging, obesity, exercise and neurological disorders, where their effects are achieved through intercellular communication. Such secreted proteins are used both as disease markers and as important indicators for the clinical diagnosis of different disease periods (24, 25). Annotation of senescence modifiers by secretory proteomics analyses captures changes in factors in serum, with more dynamic fluctuations in the transcriptome indicating more pronounced expression of secreted protein. Changes in the composition of senescence modifiers in serum according to gender in physiological states are visualized, and secreted proteomics data in physiological states in the brain, heart and muscle associated with aging are analyzed. SEPDB collects data on aging-related proteins in plasma. SEPDB contains expression data of secreted proteins in 170 human samples of different ages. We also labeled the secreted protein in these aging-related proteins based on the SEPDB dataset. Trends in aging-related secreted proteins were fitted to age in scatter plots, and differences in trends between men and women were calculated. Thus, the phenomenon of age-related changes in secreted proteins was explored in large samples (8, 26).

Although cellular senescence is an irreversible process, it can be modified. Senescence modifiers, which include secreted proteins, chemokines, growth factors, antibodies and proteases, are increasingly recognized as key drivers in the development of many chronic diseases. Studies have shown that senescence can be induced in cultured cells by X-ray irradiation, providing a means for visualizing the development of the cellular senescence phenotype in a very short time (2 weeks). This experimental approach also enables a quantitative comparison of proteins secreted by senescent cells and annotation of changes in key senescence modifiers (24, 27). Some secreted proteins are thought to be drivers of the aging process. SEPDB provides a dataset of secreted proteins associated with aging, showing how secreted proteins change in physiological states (28).

### Secreted proteins in the exercise module

Exercise-induced metabolic changes, mediated by a combination of skeletal muscle contractile proteins and biological clock proteins, cause significant changes in the muscle transcriptome and metabolic profile. In mice, locomotor capacity varies from day to day, depending on locomotor factors and molecular components of the biological clock (29). Given this metabolic and circadian control of locomotor capacity, studying locomotion and biological clocks is particularly important for mouse transcriptomics and secretory proteomics.

Exercise-related secretory protein is involved in various metabolic pathways and the propagation of signaling functions. A proteomic analysis of these secreted proteins revealed significant enrichment for immune-related processes among these biomolecules. SEPDB annotations show a time-dependent effect of exercise factors between early and late groups, identified by pooling time-related exercise factor-related proteomics data on skeletal muscle metabolic profiles, complemented by transcriptomic analyses (30, 31). On the basis of the exercise-related proteins reported in the literatures, these data were overlapped with SEPDB secreted protein data, and a total of 554 exercise-related secreted proteins were screened. Twenty-one cell types with 256 exercise-associated secreted protein pairs were included in the SEPDB. The database now also features 10 tissue-associated maps that are closely related to specific cell types. SEPDB provides tissue-specific and closely related secreted protein molecules from skeletal muscle, liver and other organs that may have beneficial effects on obesity and diabetes (32).

### Implementation of the web database

The SEPDB backends are written using the R programming language, the Shiny framework (https://shiny.rstudio.com/) and SQLlite. The front-end interface is implemented using HyperText Markup Language (HTML), Cascading Style Sheets (CSS), jQuery and JavaScript. SEPDB is deployed using Centos7 via the Nginx web server (http://nginx.org). Its online version is available at https://sysomics.com/SEPDB/ and does not require authentication or registration. SEPDB supports css layout for all major browser engines, including Google Chrome, Microsoft Edge, Safari and more. All SEPDB data are available online without restriction.

## Database content and use

### Search function

The search interface allows users to search the secreted protein core dataset by official gene symbol, protein name and chromosome name and includes plasma, protein and transcriptomics validation data for secreted proteins, as well as related information in the literature, accessible through PubMed identifiers (18, 33). Search results are displayed on a page that includes evidence validating the retrieved secreted protein together with essential information such as secretory site, subcellular location and other relevant annotations found in the literature, as shown in Figure 2. Users can search by chromosome group, protein ID, keywords and core datasets, with detailed data lists and statistical tables for proteomics and transcriptomics provided in each tissue partition. Information on whether the protein is detected in exosomes, serum and/or tissue culture supernatants is included. Moreover, the protein’s specific expression location, as determined by transcriptomics, is annotated to provide insight into whether it serves as a marker for certain diseases. The search feature content display has been optimized to ensure that each validated secretory protein is logically presented alongside its relevant information. This complements the expression of secreted proteins in serum and reveals abnormal expression in various other tissues, providing information that is highly relevant to disease development and prognosis. It also provides the user with references to data that are not retrieved and may not be documented as a secreted protein by other evidence. Data on intercellular communication enable users to selectively view information about the receptor to which the secreted protein binds as a ligand, facilitating a deeper understanding of the target and secretory pathway associated with the secreted protein.

### Browse function

To facilitate retrieval, we curated secreted protein datasets obtained from the literature and those predicted by signal peptides and constructed different models of secreted protein datasets, extending them to include biological markers of aging and chronic diseases such as cancer. SEPDB establishes an axis of disease-protein molecular relationships by comparing the core datasets with other disease metabolites and by accessing the HPA and UniProt datasets. Users can view metabolite and histological information on protein families during human development in data table format and refer to secreted protein-targeted tissue receptor expression networks for precise drug targeting (34, 35). The core dataset contains transcriptomic and proteomic data from serum, exosomes and tissue culture media; curated literature data on secreted proteins and data on subcellular localization from the UniProt database; and database resources such as SPD, HPA and other validated databases for secreted proteins and serum biomarker data (36).

### Validation and function of secreted proteins

SEPDB screens secreted proteins for inclusion and interprets annotated information, functional mechanisms and disease relevance of validated secreted proteins. As shown in Figure 2, SEPDB interprets sources of evidence for secreted proteins and expands the dataset of secreted proteins. Transcriptomic and proteomic data on secreted proteins are complemented by screening for tissue-associated proteins. Partial validation of a secreted protein revealed its role as a senescence modifier, illuminating dynamic alterations in the protein’s function. In another example, SEPDB illuminates the metabolic patterns of exercise-related secretory proteins over time, updating the distribution patterns of secretory proteins in different states throughout the day.

### Visualization

SEPDB not only offers visualization of each secreted protein entity but also provides more comprehensive information about secreted proteomics when given a list as input. SEPDB visualizes public epigenetic data on secreted proteins using the embedded WashU Epigenome Browser, which allows users to visualize search data on a single page and connect genomic coordinates to multi-resolution 3D models of chromatin. This feature helps users query the chromatin position and functional structure of the gene corresponding to the secreted protein, annotate secreted protein sequence information with more module markers and visualize the functional network of secreted proteins (37).

### Data download function and web display

SEPDB’s integrated data are accessible and downloadable online, serving as a valuable resource for retrieving datasets and evaluating evidence that allows users to reanalyze the sorted data according to their specific requirements. The SEPDB core dataset amalgamates data from multiple databases, adding additional rigor to the validation standards of secreted proteins and providing baseline data for a variety of model predictions—all freely available online. The download interface includes core data spanning a wide range of model organisms and provides user the ability to perform statistical analyses on these data from a holistic perspective.

**Figure 3.**
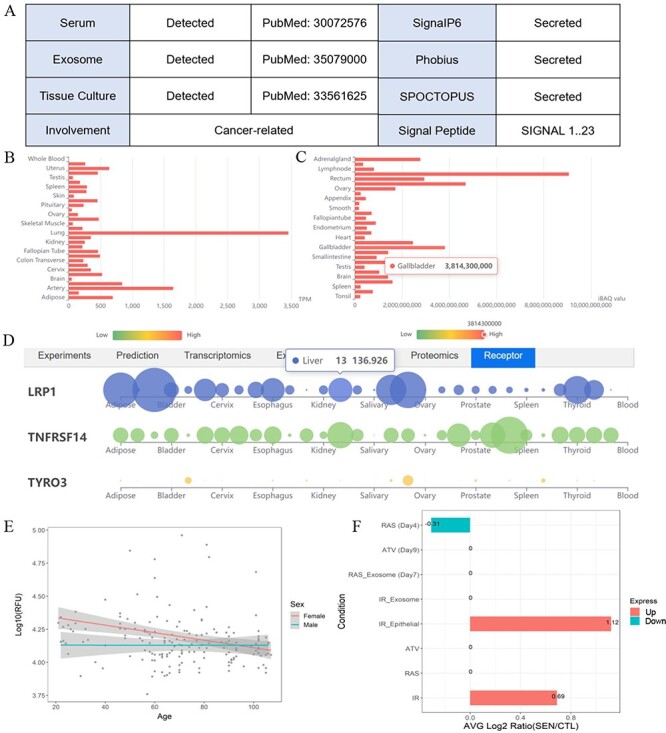
One example illustrates the multiple validation channels SEPDB provides for secreted proteins, helping to uncover protein function. (**A**) Secreted proteins validate evidence information. (**B**) Tissue-specific transcript data of alpha-2 macroglobulin. (**C**) Tissue-specific proteomic data of alpha-2 macroglobulin. (**D**) Alpha-2 macroglobulin receptor tissue-specific expression network. (**E**) Expression of alpha-2 macroglobulin in serum changed with age in 170 individuals. (**F**) Senescence-associated secretory phenotype (SASP) expression changes of alpha-2 macroglobulin after intervention under different conditions.

## Data statistics

SEPDB annotates the roles and functions of amino acids within proteins, utilizing information on secretory protein structure and predicted signal peptides. Using transcriptomics and proteomics, SEPDB maps the tissue of origin of secreted proteins, capturing secreted proteins from brain, skeletal muscle, heart, liver and adipose tissue associated with physiological states such as exercise and aging (38). Secreted proteins play an important role in intercellular communication. SEPDB delves into this by examining protein interactions to construct inter-tissue communication networks according to the information on the tissue specificity of receptors for secreted proteins. This approach aligns with that employed in previous experimental studies. SEPDB contains a dataset of 15 068 proteins from humans, rats and mice. Of these validated proteins, 8047 (53%) and an additional 4671 protein amino acid sequences were detected as secreted by forecasting tool. A total of 555 age-modifying factors that specifically correlate with receptor expression in the brain, skeletal muscle and heart were also included. In addition, proteomic information on 480 murine secreted proteins associated with the exercise duration and 456 secreted proteins related only to the level of exercise was reported.

**Table 1.** Comparison of SEPDB with related databases

	SEPDB	UniPort	HPA	NCBI	SPD
Subcellular localization	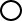	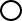	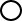	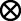	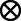
Literature	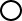	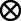	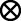	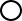	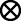
Signal peptides	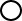	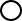	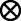	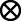	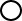
Multi-model organisms	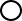	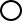	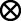	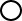	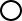
Serum	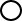	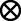	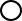	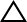	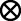
Tissue specificity	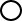	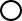	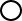	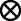	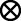
Ligand receptor	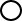	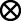	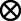	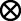	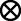
Disease development	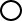	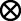	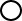	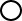	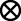

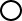
 Yes 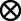 No 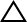 Uncertainly.

SEPDB integrates evidence for secreted proteins in rats and mice and provides core datasets from both. These datasets come not only from validated literature on plasma but also from SPD and UniProt databases and predictive validation datasets. Users can promptly query similarities and differences in the expression of secreted proteins in different tissues. SEPDB includes references for data on various biological models of secreted proteins and provides gene translation programs for different biological models in the National Center for Biotechnology Information-RefSeq database. To account for secretion levels in different model ogranisms, SEPDB has expanded its secretion set data to include references to experimental mouse and rat data.

## Case studies

SEPDB provides validation data of a secreted protein in tissue culture medium, relevant literature validation information in exosomes and serum, prediction results of the latest models and validation evidence in other databases. Users can also explore whether the expression changes of this protein in different states such as aging and exercise are markers of diseases such as tumors and inflammation. SEPDB provides the specific function of this secreted protein, as well as related receptor information on tissue-specific networks. Alpha2 macroglobulin (A2M) is taken as an example to show how users can benefit from SEPDB to explore the secreted potential and functions of alpha-2 macroglobulin. A2M and its encoded protein, alpha-2 macroglobulin, possess diverse functionalities. To start the exploration of alpha-2 macroglobulin, users can search the corresponding gene A2M with checkbox ‘Human’ checked. Utilizing the A2M gene within SEPDB enables the retrieval of various validation data, including data from tissue culture medium, documented literature reports in exosomes and serum, as well as multiple high-scoring model predictions. These datasets collectively provide robust support, with a validation score of 5, strongly indicating the classification of A2M as a secreted protein (Figure 3A). Relevant literature also underscores A2M’s secretion into the bloodstream, underscoring the data integration capabilities of SEPDB in aiding users in the discovery of potential secreted proteins. SEPDB also encompasses protein-specific transcriptomic data of corresponding receptor to facilitate a more comprehensive understanding of the target protein (Figure 3B–D). Moreover, SEPDB has collated experimental and omics data relating to A2M in the contexts of exercise and aging, revealing a declining trend in the alpha-2 macroglobulin protein during the aging process, with significant disparities evident in ionizing radiation-induced aging phenotypes (Figure 3E and F). Through the integration of diverse experimental and computational data, SEPDB strongly suggests that A2M is highly likely to be a secreted protein, offering multidimensional data to empower users in exploring the functions of A2M and its associated proteins. In summary, SEPDB provides a platform for the verification and functional display of secreted proteins.

## Discussion

Since 2003, secretory proteins have been defined as ‘secreted proteins and peptides synthesized and released by cells to perform autocrine, paracrine or endocrine functions’. Steadily increasing numbers of secretory proteins have been found to regulate organ function, reshaping our understanding of the relationship between protein factors and disease. Modern molecular biology recognizes that secreted proteins are important for maintaining or monitoring the health of the body. By aggregating large volumes of secreted protein data, SEPDB offers a user-friendly platform for biologists and clinicians to use in exploring the functional structure of secreted proteins, their changes in serum and immunological associations, as well as the diversity of their signaling receptors and interactions between tissues. Although navigating the structural features of proteins and the pathways involved in their secretion can be a challenge, we have endeavored to make SEPDB as accessible as possible.

Comparing with existing protein annotation database such as UniProt and HPA, SEPDB focuses on the secreted proteins with integrated publications and multi-omics data (Table 1). The SPD database, which is specifically designed for the prediction of secreted proteins, relies on the traditional hidden Markov model to classify predicted secreted proteins. Notably, however, SPD provides relatively little evidence to verify the secreted proteins and does not elucidate the specific functions or important roles of the secreted proteins. UniProt is a recognized biological macromolecular database containing large amounts of protein data, a rich annotated manual of proteins, and the potential to explore functional pathways. However, it is limited in that it does not allow further exploration of secreted proteins, their interaction mechanisms or signaling components between proteins and tissues. The Human Protein Atlas (HPA) contains only part of the human secretion dataset. However, it focuses only on a subset of human proteins, hindering the comprehensive exploration of the biological basis of secreted proteins. In contrast, SEPDB is a database that combines functional information with structural prediction to interpret secreted proteins, develop unique validation pipelines for secreted proteins, provide methods for analyzing the receptor specificity of secreted proteins, elucidate the origin and function of secreted proteins through transcriptomics and proteomics and identify new forms of signaling molecules in serum during exercise or aging. SEPDB explores the functional diversity of biological macromolecules by improving the process for verifying secreted proteins, but it is not limited to this purpose. Unlike SPD, UniProt and other databases, SEPDB offers comprehensive analyses now and will provide more module functionality in future versions. In the future versions, SEPDB will focus on changes in secreted proteins in different tissues under different conditions, such as exercise and aging.

In summary, SEPDB serves as a validated database for the secretory proteomics across multiple organism models, facilitating the study of protein molecule functions in both biological and clinical contexts.

## Data Availability

SEPDB is freely available online at https://sysomics.com/SEPDB/.
