# Correction to: SEPDB: a database of secreted proteins

**DOI:** 10.1093/database/baae024

**Published:** 2024-03-26

This is a correction to: Ruiqing Wang, Chao Ren, Tian Gao, Hao Li, Xiaochen Bo, Dahai Zhu, Dan Zhang, Hebing Chen, Yong Zhang, SEPDB: a database of secreted proteins, Database, Volume 2024, 2024, baae007, https://doi.org/10.1093/database/baae007

In the originally published version of this article, the symbol of equal contribution ‡ was erroneously omitted for authors Chao Ren and Tian Gao.

This has been corrected.

